# Osteoblasts from osteoarthritis patients show enhanced susceptibility to Ross River virus infection associated with delayed type I interferon responses

**DOI:** 10.1186/s12985-014-0189-9

**Published:** 2014-11-19

**Authors:** Weiqiang Chen, Suan-Sin Foo, Rachel W Li, Paul N Smith, Suresh Mahalingam

**Affiliations:** Emerging Viruses and Inflammation Research Group, Institute for Glycomics, Griffith University, Gold Coast Campus, QLD 4222 Australia; Trauma and Orthopaedic Research Unit Laboratory, The Medical School, The Australian National University, Garran Rd, Canberra, ACT 2601 Australia; Department of Orthopaedic Surgery, Trauma and Orthopaedic Research Unit, The Canberra Hospital, Canberra, ACT 2605 Australia

**Keywords:** Ross River virus, Human osteoblasts, RANKL/OPG ratio, Osteotropic factors

## Abstract

**Background:**

Arthritogenic alphaviruses such as Ross River virus (RRV) and chikungunya virus (CHIKV) have caused widespread outbreaks of chronic polyarthritis. The inflammatory responses in alphavirus-induced arthritis and osteoarthritis (OA) share many similar features, which suggests the possibility of exacerbated alphavirus-induced bone pathology in individuals with pre-existing OA. Here, we investigated the susceptibility of osteoblasts (OBs) from OA patients to RRV infection and dissected the immune mechanisms elicited from infection.

**Methods:**

Primary hOBs obtained from trabecular bone of healthy donors and OA patients were infected with RRV. Infectivity and viral replication were determined using flow cytometry and plaque assay, respectively. Real-time PCR was performed to determine expression kinetics of type I interferon (IFN)-related immune mediators and osteotropic factors.

**Results:**

OA hOBs showed enhanced RRV infectivity and replication during infection, which was associated with delayed induction of IFN-β and RIG-I expression. Enhanced susceptibility of OA hOBs to RRV was associated with a more pronounced increase in RANKL/OPG ratio and expression of osteotropic factors (IL-6, IL-1β, TNF-α and CCL2) in comparison to RRV-infected healthy hOBs.

**Conclusions:**

Delayed activation of type I IFN-signalling pathway may have contributed to enhanced susceptibility to RRV infection in hOBs from OA patients. RRV-induced increases in RANKL/OPG ratio and expression of osteotropic factors that favour bone resorption, which may be exacerbated during osteoarthritis. This study provides the novel insight that osteoarthritis may be a risk factor for exacerbated arthritogenic alphaviral infection.

## Background

Ongoing global warming and increased rainfall variability globally have provided extensive breeding grounds for arthropod vectors. Infection rates of arthropod-borne alphaviruses such as Ross River virus (RRV) and chikungunya virus (CHIKV) are currently rising in different geographic regions of the world [1]. Debilitating polyarthralgia/polyarthritis is the clinical hallmark of RRV and CHIKV infection, which typically affects ankle joints, knees and peripheral joints [2-4]. The onset of RRV disease (RRVD) and chikungunya fever (CHIKF) can be sudden and debilitating, with the level of disability comparable to other forms of arthritis such as osteoarthritis (OA) or chronic rheumatoid arthritis (RA) [5].

Prolonged manifestations of chronic RRVD and CHIKF, including persistent polyarthritis and/or polyarthralgias, occur in approximately 40% of infected individuals [6]. Pro-inflammatory immune mediators, including interleukin (IL)-6, IL-1, tumor necrosis factor-α (TNF-α) and monocyte chemotactic protein-1 (MCP-1; or CCL2) have been shown to contribute to the progression of alphaviral disease [7-9]. Interestingly, these pro-inflammatory cytokines are also known as osteotropic factors that modulate the bone remodelling system.

The human bone remodelling system requires the coordinated action of bone-resorbing osteoclasts (OCs) and bone-forming osteoblasts (OBs), which are together termed the basic multicellular unit (BMU), to maintain bone homeostasis. OBs are involved in bone formation and mineralisation and orchestrate cell signalling pathways required for local control of OC differentiation [10]. In most arthritic conditions, OCs have been identified as the principal cells responsible for bone erosions [11]. Enhanced production of key OC differentiation factors such as receptor activator of nuclear factor (NF)-κβ ligand (RANKL), IL-6, IL-1 and TNF-α by OBs can stimulate osteoclastogenesis [10,12]. To maintain bone homeostasis, osteoclastogenesis is kept in check by OB-secreted osteoprotegerin (OPG), which inhibits OC differentiation by acting as a soluble decoy receptor for RANKL. Hence, an increase in RANKL/OPG ratio is associated with increased OC activity, which may result in osteolytic pathologies [13-15].

Clinical evidence of bony erosions has been reported in the joints of CHIKV-infected patients [16], providing evidence that alphavirus-induced disease can result in erosive arthritis. Recently, we reported pathologic bone loss during acute RRV infection in an acute RRVD mouse model, which is consistent with the increased tartrate-resistant acid phosphatase 5b (TRAP5b) level and RANKL/OPG ratio in serum of RRV patients. RRV infection of primary human osteoblasts (hOBs) also leads to an increased RANKL/OPG ratio and robust production of pro-inflammatory cytokines IL-6 and CCL2 [17].

OA, also known as degenerative joint arthritis, is the most common form of arthritis. It is characterized by degradation of cartilage and inflammation of the synovium and affects millions of people worldwide, with an estimate of over 1.4 million people affected in Australia alone [18]. Pro-inflammatory cytokines such as IL-6, IL-1, TNF-α and CCL2 are involved in OA severity: this cytokine profile has a striking resemblance to inflammatory responses elicited in alphavirus-induced arthritis [19]. The shared cytokine profile between alphavirus-induced arthritis and OA highlights a potential for exacerbation of alphavirus-induced bone loss in individuals with pre-existing OA [3,20,21].

In this study, we investigated the potential of underlying OA to influence alphavirus infection. RRV infection of OA hOBs led to enhanced viral infectivity and replication, accompanied by delayed type I IFN responses. RRV-induced pro-inflammatory immune responses were increased in OA hOBs. The imbalance in the RANKL/OPG axis that results from RRV infection was further perturbed in hOBs from OA patients, suggesting OA as a predisposing risk factor for alphavirus-induced bone pathology.

## Results

### Enhanced RRV infectivity in primary hOBs of OA patients, modulated through delayed type IFN signalling pathway

**Figure 1:**
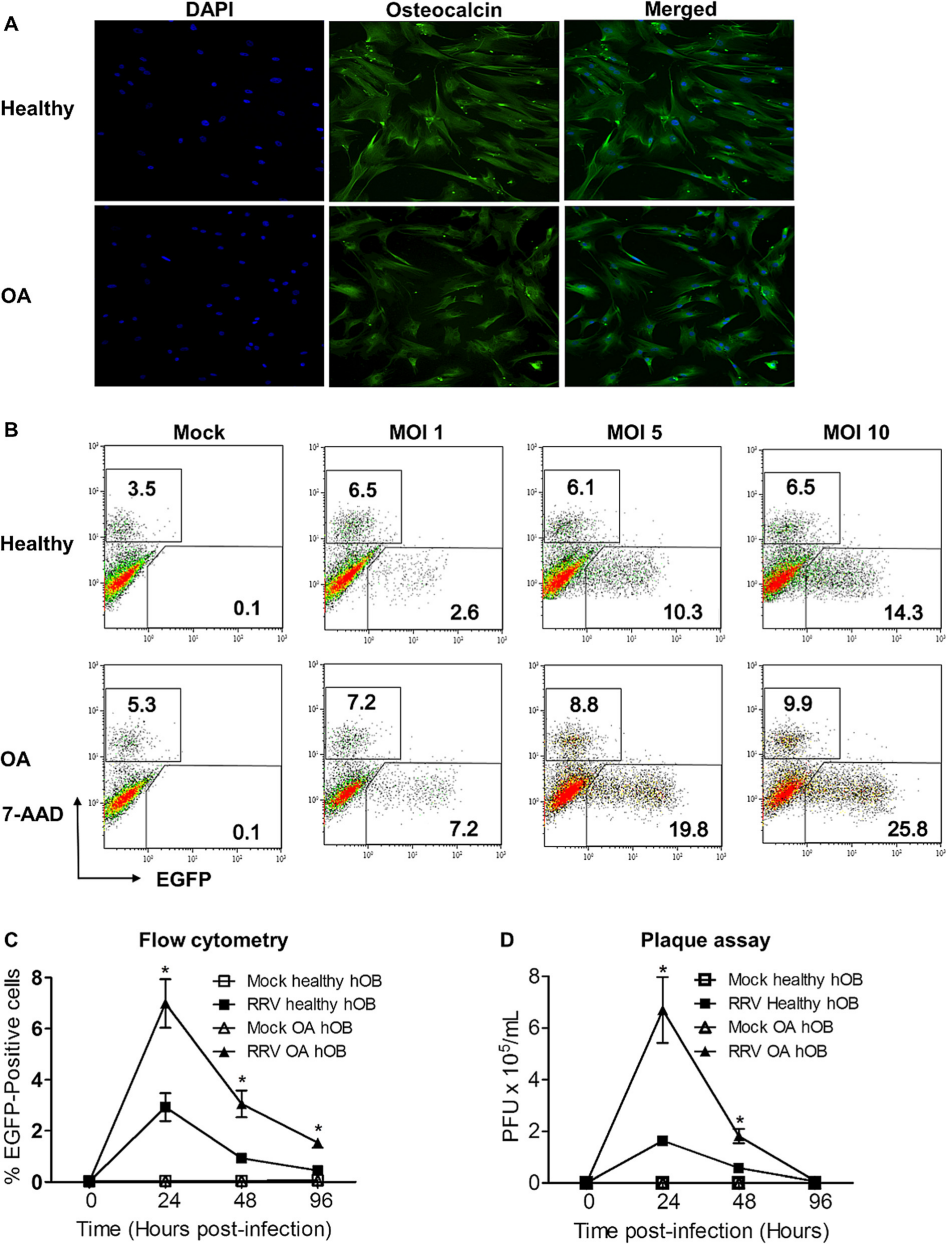
**Enhanced RRV replication in primary hOBs of OA patients. (A)** Phenotypic characterization of primary hOBs. Primary hOBs were fixed and stained for osteocalcin (green) and nuclei (blue), and visualized by confocal microscopy. Magnification, ×20. Images are representative of 2 independent experiments. Primary hOBs were infected with RRV-EGFP at MOI 1 and cells and supernatant were harvested at different time points. **(B)** Dose-dependent infection of hOBs. Cell harvested at 24 hpi were fixed, stained for 7-AAD, and subjected to FACS analysis. FACS scatter plots are representative of the data obtained for 3 independent experiments. **(C)** Quantification of percentage EGFP^+^ cells after infection. **(D)** Supernatant was harvested and RRV titre was determined by plaque assay on Vero cells. Data (*n* =4) are presented as mean ± SEM. *P <0.05, two-way ANOVA, Bonferroni post-test.

To investigate if underlying OA has any effect on viral replication during RRV infection, primary hOBs cultured from trabecular bones of healthy donors and OA patients were used. The cultured primary hOBs were phenotypically characterized using osteocalcin, an OB marker, prior to RRV infection (Figure 1A). To determine the infectivity of these primary hOB cultures, they were infected with RRV-EGFP at a series of multiplicity of infection (MOI) from 1 to 10. Infectivity of both healthy and OA hOBs was demonstrated in a dose-dependent manner (Figure 1B). The kinetics of RRV replication in the primary hOBs was further determined through a 96 h time-course infection, with cells and supernatant harvested at 24, 48 and 96 hours post-infection (hpi) for FACS analysis and plaque assays. Peak RRV replication was observed at 24 hpi for both healthy and OA hOBs, with a subsequent decline over the next 72 h. Interestingly, FACS analysis of the harvested cells revealed significantly enhanced RRV infectivity in OA hOBs at 24, 48 and 96 hpi (Figure 1C). Consistent with the enhanced infectivity in OA hOBs, higher levels of virus were also recovered from the supernatant of infected OA hOBs at 24 and 48 hpi, compared to healthy hOBs (Figure 1D).

**Figure 2:**
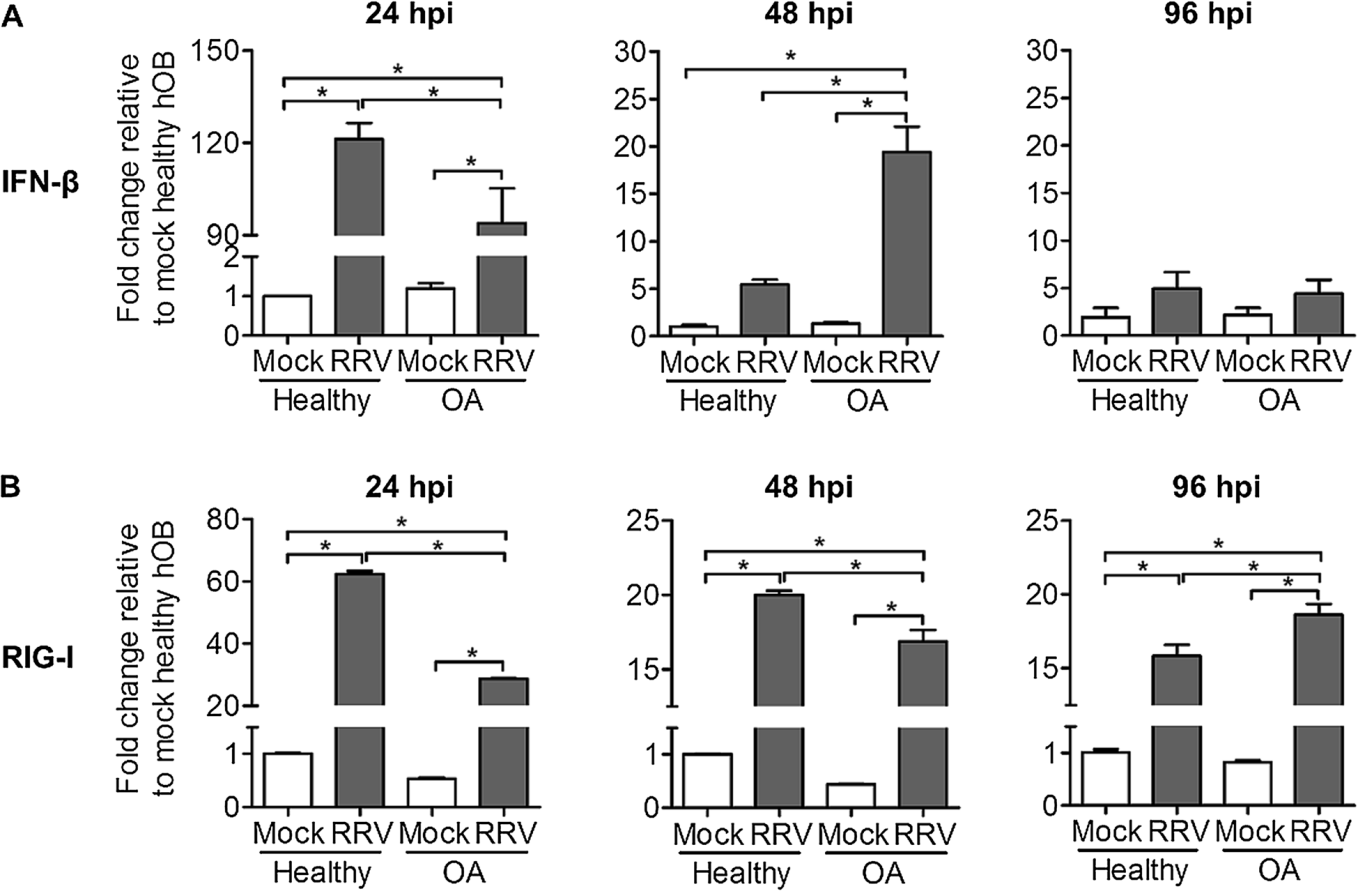
**Delayed IFN-signalling pathway in primary hOBs of OA patients during RRV infection.** Primary hOBs were infected with EGFP-RRV at MOI 1 and cells were harvested at different time points for RNA extraction. Transcriptional profiles of **(A)** IFN-β and **(B)** RIG-I were determined using qRT-PCR. Data are normalized to HPRT and shown as fold expression relative to healthy mock-infected group. Data (*n* =4) are presented as mean ± SEM. *P <0.05, one-way ANOVA, Tukey’s post-test.

The type I IFN signalling pathway serves as the first line of host defence against pathogen invasion. We sought to determine the involvement of type I IFN in the contribution of enhanced RRV infectivity and replication in OA hOBs. Infected primary hOBs were harvested at 24, 48 and 96 hpi for determination of IFN-β and RIG-I mRNA expression (Figure 2). At the peak viral replication of 24 hpi, RRV-infected OA hOBs had significantly lower transcriptional induction of both IFN-β and RIG-I compared to RRV-infected healthy hOBs. The induction of IFN-β and RIG-I in RRV-infected OA hOBs gradually overtook that of RRV-infected healthy hOBs at 48 and 96 hpi, respectively. Together, these data suggest that enhanced susceptibility and viral replication in OA hOBs may be modulated through delayed induction of type I IFN.

### Underlying OA further perturbs bone homeostasis after RRV infection

**Figure 3:**
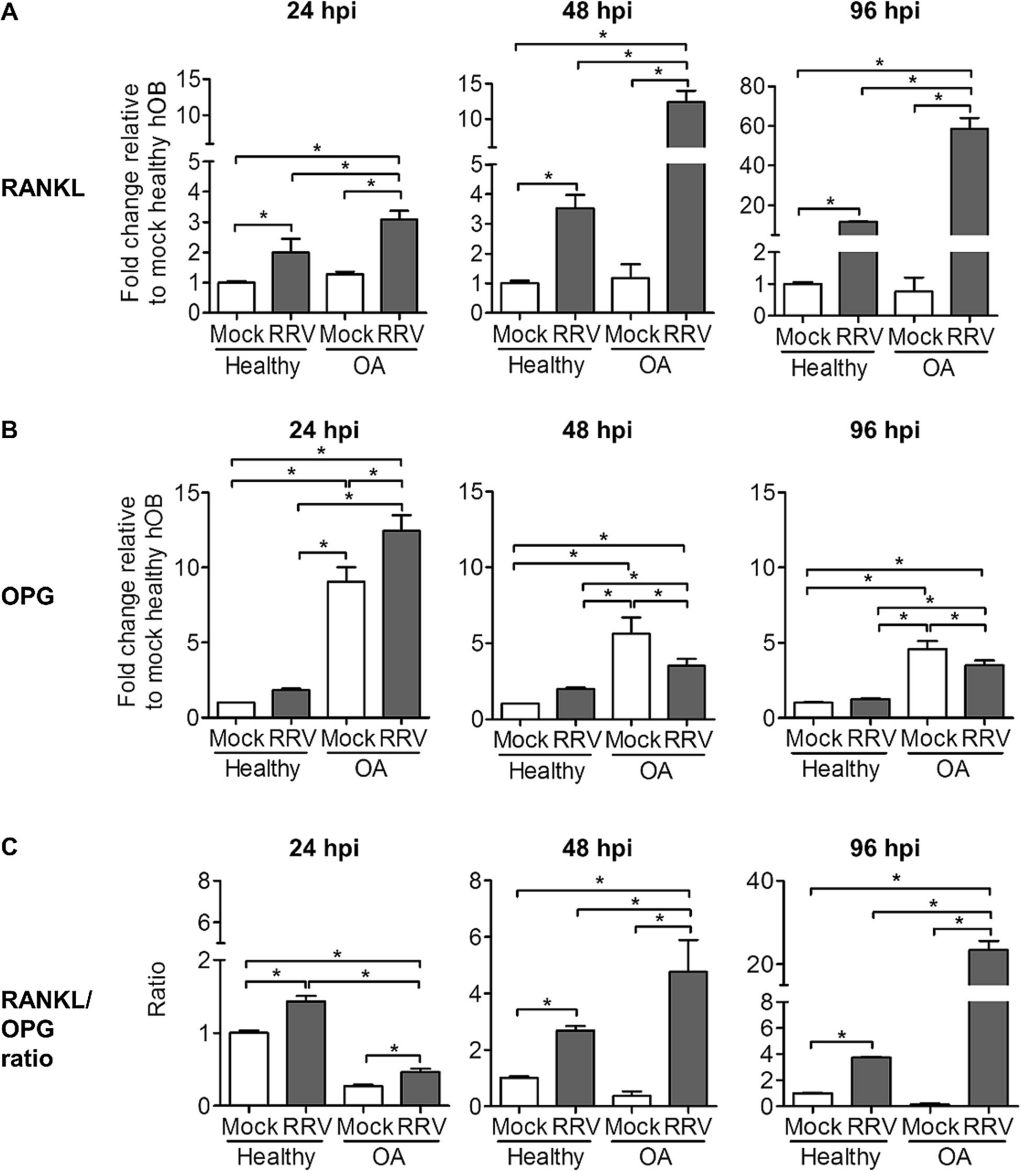
**Underlying OA condition further perturbs RANKL/OPG ratio after RRV infection in primary hOBs.** Primary hOBs were infected with EGFP-RRV at MOI 1 and cells were harvested at different time points for RNA extraction. Transcriptional profiles of **(A)** RANKL and **(B)** OPG were determined using qRT-PCR. **(C)** RANKL/OPG ratio is shown. Data are normalized to HPRT and shown as fold expression relative to healthy mock-infected group. Data (*n* =4) are presented as mean ± SEM. *P <0.05, one-way ANOVA, Tukey’s post-test.

Alphavirus-induced arthritis and OA are inflammatory arthritides that have been associated with bone disorders [17,22]. Recently, we demonstrated that RRV infection can elicit bone loss through perturbed OB function. Therefore, we investigated the effect of RRV infection on bone regulatory function of OA hOBs by measuring RANKL and OPG, which are the fundamental elements of bone homeostasis. Briefly, primary hOB cultures were infected at MOI 1 and harvested at 24, 48 and 96 hpi for determination of RANKL and OPG transcript expression. RANKL expression in OA hOBs was significantly higher at all time points than in healthy hOBs after RRV infection. A drastic increase in RANKL expression of ~20-fold between 24 to 96 hpi was observed in RRV-infected OA hOBs, compared to ~5-fold increase for RRV-infected healthy hOBs. No significant difference in RANKL expression was observed between mock-infected healthy and OA hOBs (Figure 3A). Higher levels of OPG expression were detected in mock-infected OA hOBs compared to mock- or RRV-infected healthy hOBs. After RRV infection, OA hOBs demonstrated higher expression of OPG compared to healthy infected hOBs at all tested time points. No significant changes in OPG expression were observed between mock- or RRV-infected healthy hOBs at all tested time points. However, at 48 and 96 hpi, OPG levels were significantly lower in the RRV-infected OA hOBs compared to mock-infected OA hOBs (Figure 3B). Thus, RRV infection resulted in a markedly elevated RANKL/OPG ratio in both healthy and OA hOBs compared to mock-infected groups. Interestingly, OA hOBs exhibited a significantly higher RANKL/OPG ratio than healthy hOBs after RRV infection at 48 and 96 hpi (Figure 3C), suggesting that underlying OA may exacerbate RRV-induced bone pathology by further increasing the RANKL/OPG ratio.

### Increased expression of osteotropic factors in primary hOBs of OA patients after RRV infection

**Figure 4:**
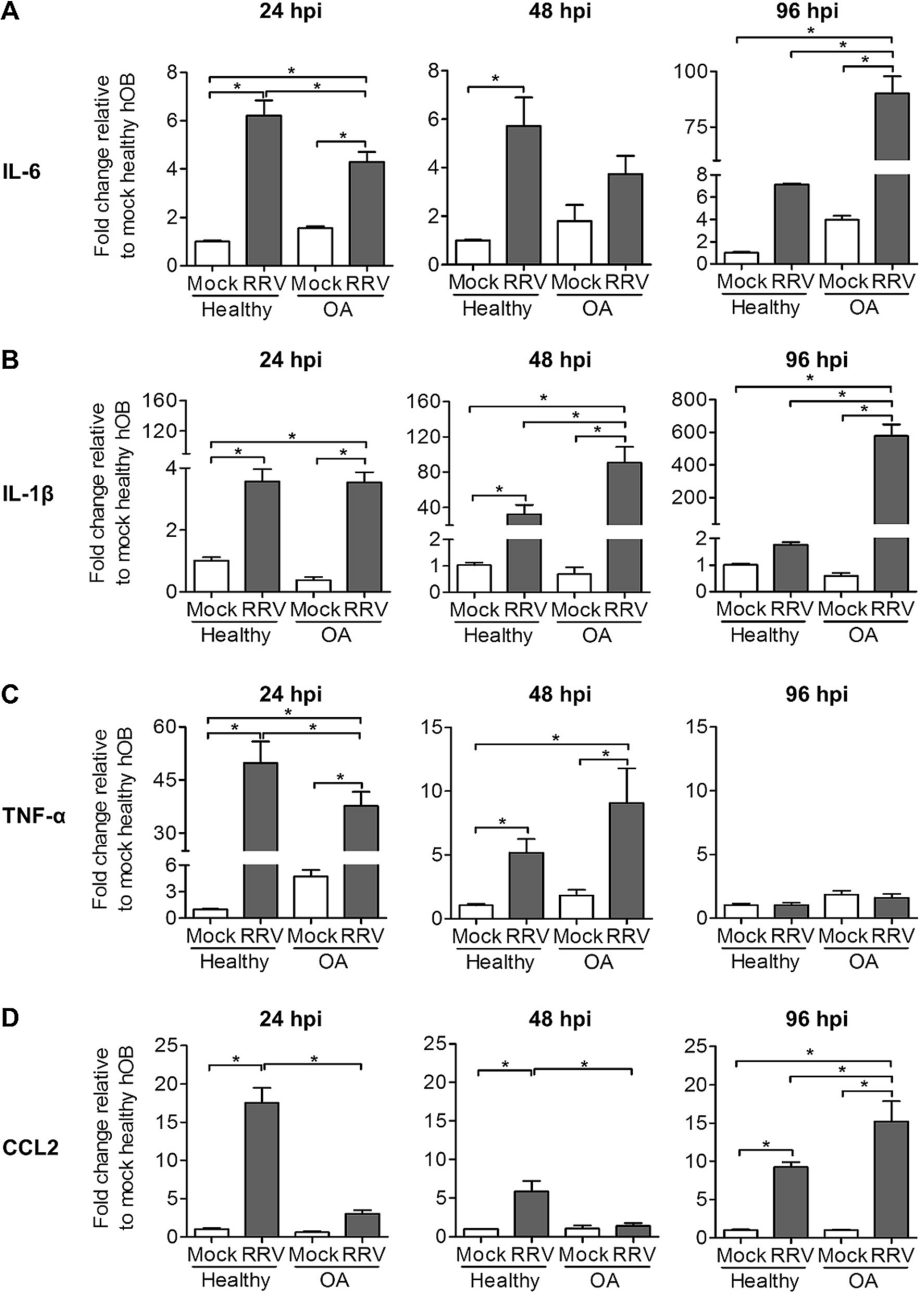
**Enhanced expression of osteotropic factors in primary hOBs of OA patients after RRV infection.** Primary hOBs were infected with EGFP-RRV at MOI 1 and cells were harvested at different time points for RNA extraction. Transcriptional profiles of osteotropic factors **(A)** IL-6, **(B)** IL-1β, **(C)** TNF-α and **(D)** CCL2 were determined using qRT-PCR. Data are normalized to HPRT and shown as fold expression relative to healthy mock-infected group. Data (*n* =4) are presented as mean ± SEM. *P <0.05, one-way ANOVA, Tukey’s post-test.

Pro-inflammatory mediators such as IL-6, IL-1β, TNF-α and CCL2 contribute to alphaviral disease pathogenesis. Interestingly, these immune mediators also function as osteotropic factors, with a pivotal role in modulating bone remodelling [23,24]. Transcriptional analysis of these osteotropic factors was performed to assess their expression kinetics. IL-6 expression was up-regulated at all tested time points after RRV infection in both healthy and OA hOBs. At 24 and 48 hpi, RRV infection of OA hOBs elicited consistently lower levels of IL-6 transcription compared to healthy hOBs, but its expression was increased at 96 hpi (Figure 4A). Early induction of IL-1β expression was observed in both healthy and OA hOBs after RRV infection at 24 hpi, which peaked at 48 hpi, with ~30-fold increase for RRV-infected healthy hOBs and ~90-fold increase for RRV-infected OA hOBs. At 96 hpi, the RRV-induced expression of IL-1β in healthy hOBs declined, while transcription continued to increase dramatically to ~600-fold change in RRV-infected OA hOBs (Figure 4B). Elevated expression of TNF-α was detected at 24 hpi in RRV-infected healthy and OA hOBs, but to a lesser extent for the OA group. The induction of TNF-α declined rapidly at 48 hpi and reverted to basal level by 96 hpi (Figure 4C). CCL2 expression was highly up-regulated in healthy hOBs after RRV infection at 24 hpi and decreased gradually over the next 72 h. In contrast, RRV-induced CCL2 expression in OA hOBs only commenced at 96 hpi (Figure 4D). Collectively, the elevated expression of these pro-inflammatory/osteotropic factors at later stages of infection may stimulate RANKL-mediated osteoclastogenesis.

## Discussion

Alphavirus-infected patients suffering from pre-existing joint disorders such as OA have been reported to be at possible increased risk of severe joint arthralgia and delayed remission [3,16,20,25,26]. However, the mechanism behind prolonged and exacerbated disease in patients with underlying arthritis has not been elucidated. Our previous findings highlighted that alphaviruses possess tropism for murine bones and that OB-lineage cells have potential roles in the pathogenesis of alphaviral arthritis [17,27,28]. This study was undertaken to investigate the susceptibility and response of primary hOB from OA patients to arthritogenic RRV infection and to analyse if infection can exacerbate hOB osteoclastogenic potential.

Primary hOBs from OA patients were significantly more susceptible to RRV infection and the virus replicated at higher levels compared to hOBs from healthy donors. Alphaviruses are susceptible to type I IFNs, which was elicited in response to RNA viruses by the cytoplasmic RNA sensor RIG-I [29-32]. A possible explanation for the enhanced infection of OA hOBs was the lower expression of RIG-I and IFN-β compared to healthy cells at peak infection (24 hpi), suggesting a delayed IFN-signalling pathway in OA hOBs.

Bone cells such as OB play a pivotal role in bone remodelling, with primary *role* in bone formation and regulation of OC bone resorption through the OPG/RANKL/RANK system [33,34]. Cells of OB lineage express both RANKL and OPG, which are key osteotropic factors in regulating bone remodelling; a higher RANKL/OPG ratio favours osteoclastogenesis [13-15]. At basal levels, we found a lower RANKL/OPG ratio in OA hOBs than healthy hOBs. This is in agreement with previous studies showing that OA patients have a lower RANKL/OPG ratio, suggesting reduced osteoclastic activity [35,36]. In contrast to the lower RANKL/OPG ratio in OA hOBs, the ratio in these cells was significantly increased after 96 h RRV infection compared to healthy infected cells, highlighting the potential of OA predisposed to exacerbated alphavirus-induced bone pathology.

Pro-inflammatory/osteotropic factors are crucial in both bone remodelling and in the pathogenesis of alphavirus infection. For example, IL-6 is highly expressed in affected joints during alphavirus disease [37] and is proposed to be an index of CHIKV disease severity [8,38]. Recently, we showed that IL-6, IL-1β, TNF-α and CCL2 are highly expressed in RRV-infected hOBs of healthy donors and that these infected cells are crucial in mediating RRV-induced bone loss through the up-regulation of RANKL/OPG ratio [17]. Similarly, we observed up-regulation of IL-6, IL-1β and TNF-α in OA hOBs at peak infection (24 hpi) in our study. Although RRV-induced IL-6, TNF-α and CCL2 were lower in the OA hOBs compared to infected controls during early infection, the expression of these osteotropic factors in OA hOBs, with the exception of TNF-α, was significantly elevated at 96 hpi. Hence, the increased expression of IL-6, IL-1β and CCL2 at later stages of infection may act to promote osteoclastogenesis. This is supported by the high RANKL/OPG ratio, a well-established index for osteoclastogenesis, in OA hOB after 96 hpi compared to infected controls.

Our *in vitro* findings are the first to demonstrate increased susceptibility of primary OA hOBs to RRV infection, which may contribute to disease exacerbation in patients with underlying inflammatory arthritis.

## Conclusions

These exciting results clearly indicate that the response of ‘arthritic’ osteoblasts to RRV infection differs from normal osteoblasts; they are more susceptible to infection possibly through delayed type I IFNs response and produce higher levels of IL-6, IL-1β, CCL2 and TNF-α. At later stages of infection, RRV-infected OA hOBs showed significantly increased IL-6, IL-1β and CCL2 expression compared to healthy infected controls. The increase in these pro-inflammatory/osteotropic factors by OA hOBs may act to recruit monocytic OC precursor cells to sites of infection and to enhance the expression of RANKL in RRV-infected hOBs. These delayed pro-inflammatory responses resulted in an increased RANKL/OPG ratio, which can lead to increased osteoclastogenesis. Our novel findings highlight a possible OB-mediated mechanism to explain exacerbations of joint arthralgia and prolonged disease severity after RRV infection in patients with underlying OA.

## Methods

### Viruses

Stocks of RRV strain T48 (RRV-T48) were generated from the full-length T48 cDNA clone [39]. Stocks of RRV that expressed enhanced green fluorescent protein (RRV-EGFP) were generated by inserting a second RRV 26S promoter sequence at the 3′ end of the viral genome, followed by the coding sequence for EGFP (kindly provided by Dr Mark Heise, University of North Carolina). All titrations were performed by plaque assay on Vero cells as described previously [40].

### Primary cell cultures

Primary human OB (hOB) cultures were obtained from trabecular bone specimens of 7 healthy individuals (4 males, 3 females), aged 50–60 years. Healthy hOBs were obtained from individuals undergoing orthopaedic operations for causes unrelated to arthritis and osteoporosis (such as ligament reconstruction or trauma cases). OA trabecular bone specimens were obtained from femur neck of 5 patients (3 males, 2 females) undergoing primary or revision surgery. The patients ranged in age from 50–60 years and were diagnosed with OA according to the World Health Organisation (WHO) criteria, mainly based on clinical presentation, radiographic findings. All human specimens were collected with the permission of the Human Research Ethics Committee of Australian National University (human ethics approval-ETH-9-07-865) and Australian Capital Territory (ACT) Health, together with informed patient consent. The exclusion criteria for recruiting hOB donors was (i) taking immune suppressant/stimulating drugs, cytokine, or IFN therapy in the 3 months before surgery; (ii) treatment with anabolic agents; and (iii) low quality of the bone fragment, socio-economic or ethic characteristics and age not matching with the healthy group, in the opinion of the investigators, could compromise the study. Briefly, the bone fragments were washed with PBS and cultured in α-MEM (Sigma-Aldrich) supplemented with 10% HI-FCS, 1% antibiotic solution, 100 μM ascorbic acid, 20 mM Hepes, and 2 mM L-glutamine at 37°C with 5% CO_2_. After 2 weeks, outgrowing cells surrounding bone fragments were cultured and designated as “passage 1.” The hOBs cultured in our laboratory were able to proliferate in vitro for up to five passages but were used at passage 2 in all experiments.

### Phenotypic characterisation of primary human osteoblasts (hOBs)

To characterise cultured hOB, osteocalcin expression was determined by staining hOB with primary mouse anti-human osteocalcin monoclonal antibodies (R&D Systems, Minneapolis, USA) and Alexa-Fluor 488 (AF488) goat anti-mouse IgG (Invitrogen, Melbourne, Victoria, Australia), followed by DAPI staining and visualisation using an FV1000 confocal microscope (Olympus, Australia).

### In vitro RRV infection

Primary hOBs were seeded in 24-well plates at a density of 1 × 10^5^ cells per well overnight. Cells were then infected with RRV-EGFP at a multiplicity of infection (MOI) of 1. After 1 h, cells were washed twice and cultured in fresh α-MEM with 10% FCS at 37°C with 5% CO_2_. At appropriate time points, culture medium was collected for plaque assay and cells were harvested for RNA extraction.

### Flow Cytometry (FACS)

Cells were trypsinsed and washed twice in wash buffer (PBS-2% FCS). Cells were then stained with 7-AAD (7-aminoactinomycin) (eBioscience, San Diego, CA) at room temperature for 10 min. Following 7-AAD staining, EGFP expression in RRV-infected cells was analysed using a CyAn ADP flow cytometer and Kaluza software (Beckman Coulter).

### Plaque assays

Vero cells were seeded into 24-well plates at a density of 1 × 10^5^ cells per well and cultured to confluence. Serially diluted samples (10^−1^ to 10^−6^) were added to cell monolayers and incubated for 1 h at 37°C in a 5% CO_2_ incubator. Cells were then overlaid with OPTI-MEM (Invitrogen, Melbourne, Victoria, Australia) containing 3% FCS and 1% agarose (Sigma Aldrich, Sydney, Australia) and incubated for 48 h in a 5% CO_2_ incubator. The cells were fixed with 1% formalin and plaques were visualised by staining with 0.1% crystal violet. Virus titres were expressed as plaque forming units per millilitre (PFU/mL) or PFU per gram of tissue.

### Real-time PCR (qRT-PCR)

RNA was prepared from cell pellets using TRIzol (Invitrogen, Melbourne, Victoria, Australia) according to the manufacturer’s instructions. Eluted RNA was stored at −80°C. Total RNA was measured by NanoDrop 1000 spectrophotometer (Thermo Scientific). Extracted total RNA (20 ng/μL) was reverse-transcribed using an oligo(dT) primer and reverse transcriptase (Sigma Aldrich, Sydney, Australia), according to the manufacturer’s instructions. SYBR® Green Real-time PCR was performed using 50 ng of template cDNA on a CFX96 Touch™ Real-Time PCR System in 96-well plates, using QuantiTect Primer Assay kits (Qiagen, Hilden, Germany) with the following conditions: (i) PCR initial activation step: 95°C for 15 min, 1 cycle and (ii) 3-step cycling: 94°C for 15 s, follow by 55°C for 30 s and 72°C for 30 s, 40 cycles. Amplification specificity was evaluated by a melting curve analysis of PCR products. The fold change in messenger RNA (mRNA) expression relative to mock-infected samples for each gene was calculated with the ΔΔ*Ct* method. Briefly, ΔΔ*Ct* = Δ*Ct* (RRV-infected) – Δ*Ct* (Mock-infected) with Δ*Ct* = *Ct* (gene of interest) – *Ct* (housekeeping gene - HPRT). The fold change for each gene is calculated as 2^-ΔΔ*Ct*^.

### Statistical analysis

Viral titers and flow cytometry data were statistically evaluated using two-way analysis of variance (ANOVA) with Bonferroni posttest. Real-time PCR data were analyzed using one-way ANOVA with Tukey’s posttest. Differences between groups with P <0.05 were considered significant. All data were assessed for normality using the D’Agostino-Pearson normality test before analysis with these parametric tests. All statistical analyses were performed with GraphPad Prism software, version 5.02.
